# Gestural Sequences in Wild Spider Monkeys (*Ateles geoffroyi*)

**DOI:** 10.1002/ajp.70156

**Published:** 2026-04-27

**Authors:** Eva Corral, Sara Cardoso Rodriguez, Katja Liebal, Miquel Llorente, Federica Amici

**Affiliations:** ^1^ Fundació UdG: Innovació i Formació Universitat de Girona Girona Spain; ^2^ Institute of Biology, Faculty of Life Science University of Leipzig Leipzig Germany; ^3^ Department of Comparative Cultural Psychology Max‐Planck Institute for Evolutionary Anthropology Leipzig Germany; ^4^ Comparative Minds Research Group, Departament de Psicologia, Facultat d'Educació i Psicologia Universitat de Girona Girona Spain

**Keywords:** communication, gestural sequences, platyrrhines, social context

## Abstract

To date, research on gestural communication in species other than great apes has been quite limited, especially in their natural habitat, although including a broader range of species in different settings is essential for identifying evolutionary patterns and determining whether some of the fundamental characteristics of human communication are widely shared across primates. In this study, we specifically aimed to explore the use of gestural sequences in Geoffroy's spider monkeys (*Ateles geoffroyi*), a species for which research on gestural communication remains scarce. To this end, we conducted behavioral observations, recording focal video samples of a wild group of 54 individuals. From the videos, we coded all gestures (*N* = 709), of which 182 occurred alone and 527 occurred within 125 sequences, which mostly consisted of 2–4 successive gestures. The odds of producing gestural sequences rather than single gestures were higher in the contexts of play, aggression and sexual behavior. In contrast, there was no significant effect of signaler's sex and age on the odds of producing a gestural sequence rather than a single gesture. Only three sequences (i.e., embrace‐pectoral sniff, push‐present climb, grab‐grab pull) occurred more than twice and were not mere repetitions of the same gesture type. Our results show that gestural sequences are used by platyrrhines in several different contexts, although more studies are needed to understand their function.

## Summary

- Wild Geoffroy's spider monkeys (*Ateles geoffroyi*) produced gestural sequences during natural interactions.
- The odds of producing gestural sequences rather than single gestures were higher in aggressive, playful and sexual contexts.
- The odds of producing gestural sequences rather than single gestures did not depend on signaller's sex or age, in contrast to other primate species.

## Graphical abstract

Geoffroy's spider monkeys produce gestural sequences in the wild, mostly during aggressive, playful and sexual interactions.

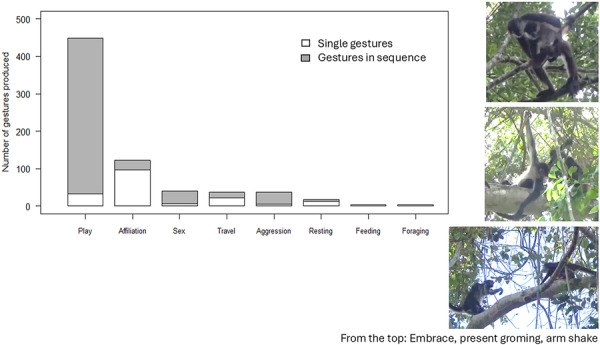

## RESUMEN

Hasta la fecha, la investigación sobre comunicación gestual en especies distintas a los grandes simios ha sido limitada, especialmente en su hábitat natural, aunque incluir un abanico más amplio de especies en diferentes contextos es esencial para identificar patrones evolutivos y determinar si las caracteristicas fundamentales de la comunicación humana son exclusivas de los grandes simios o están más ampliamente compartidas entre los primates. En este estudio, exploramos el uso de secuencias gestuales en el mono araña de Geoffroy (*Ateles geoffroyi*), una especie para la cual la investigación sobre comunicación gestual sigue siendo escasa. Con este fin, realizamos observaciones de comportamiento, registrando muestras focales de un grupo silvestre compuesto por 54 individuos. A partir de los vídeos, codificamos todos los gestos (*N* = 709), de los cuales 182 ocurrieron como gestos únicos y 527 dentro de 125 secuencias, que en su mayoría incluían entre 2 y 4 gestos. Las *odds* de producir una secuencia gestual en lugar de un gesto único fue máyor en contextos de juego, agresión o sexuales. En contraste, no se encontró ningún efecto significativo del sexo y de la edad del emisor sobre las *odds* de producir una secuencia gestual en lugar de un gesto único. Solo tres secuencias (es decir, a*brazo‐olfateo pectoral, empujón‐presentación para trepar y agarre‐tirón*) ocurrieron más de dos veces y no fueron meras repeticiones del mismo tipo de gesto. Nuestros resultados muestran que las secuencias gestuales también son utilizadas por platirrinos en diferentes contextos, aunque se necesitan más estudios para comprender su función.

**Abbreviations**
ANOVA: analysis of variance
GLMM: generalized linear mixed models

## 1 Introduction

Gestural communication in non‐human primates (hereafter primates) has been systematically studied since the 1960s (see: Cartmill and Hobaiter 2019; Graham et al. 2022; Plooij 1978; Tomasello and Call 2019; van Lawick‐Goodall 1968; 1972), with a particular focus on great apes (Bezanson and McNamara 2019). Initially, much of the interest in ape gestural communication stemmed from the hypothesis that early forms of human language could have been gestural (Arbib et al. 2008; Dediu and Levinson 2013; Fitch 2010; Levinson 2023; Tomasello 2008). In this perspective, studying apes could provide valuable insights into the evolution of human communication by revealing fundamental communicative features potentially shared with humans and highlighting evolutionary pathways that might have led to the emergence of language (Liebal et al. 2013).

Studies on primate gestural communication have provided clear evidence that great apes typically rely on rich communicative systems that include several types of gestures (or discrete, mechanically ineffective physical movements, mostly of the limbs or the head, produced in a social context, in a goal‐directed intentional way; see Genty et al. 2009; Hobaiter and Byrne 2011a, 2017; Pika 2008; Roberts et al. 2014; Tomasello et al. 1985, 1994; Tomasello and Call 2019). These different gestures convey specific meanings and show a certain degree of flexibility, being produced across various contexts and in different morphological forms (e.g., in captivity: Amici and Liebal 2025; Bard et al. 2019; Cartmill & Byrne 2010; Liebal et al. 2004; in the wild: Grund et al. 2024; Hobaiter and Byrne 2011a, 2014; Hobaiter et al. 2022; Kalan et al. 2023; Pika and Deschner 2019). While the basic forms and meanings of gestures appear largely innate, social experience allows individuals to refine their gestural communication systems, also improving their effectiveness over time (in captivity: Amici and Liebal 2022; Bard et al. 2014; in the wild: Byrne et al. 2017; Graham et al. 2018; Hobaiter and Byrne 2011a; 2011b; Roberts et al. 2019).

In recent years, the study of primate gestural communication has partially extended beyond great apes, allowing researcher to consider to what extent some of the fundamental characteristics of human language (e.g., intentionality, flexibility, compositionality: Amici and Liebal 2025; Anderson et al. 2010; Bard et al. 2019; Call and Tomasello 2007; Slocombe et al. 2022) are also shared with other species. However, many platyrrhines and various clades of nocturnal primates, remain understudied in terms of gestural communication (Gursky and Nekaris 2019; Liebal et al. 2022; Lisboa et al. 2021). This gap is significant, because including primates that are phylogenetically distant to humans or occupy distinct socio‐ecological niches is essential to infer when specific communicative traits might have emerged during our evolutionary history and which ecological or social conditions might have favored their emergence. Studies on catarrhines, for instance, have begun to reveal the existence of large communication systems characterized by intentionality and contextual flexibility (captive *Cercocebus torquatus*: Aychet et al. 2020; Maille et al. 2012; Schel et al. 2022; captive *Macaca tonkeana:* Canteloup et al. 2015; wild *Macaca radiata:* Deshpande et al. 2018; captive *Papio anubis*: Meunier et al. 2013; Molesti et al. 2020). Similar patterns have been documented in some platyrrhines, including capuchin monkeys (captive *Sapajus apella*: Defolie et al. 2015; Hattori et al. 2007), squirrel monkeys (captive *Saimiri sciureus*: Anderson et al. 2010) and spider monkeys (wild *Ateles geoffroyi*: Villa‐Larenas et al. 2024). Together, these findings suggest that some fundamental characteristics of human language and great ape gestural communication are present also in other primate species, and thus likely emerged earlier than previously thought in our evolutionary history.

One of the main hallmarks of human communication is compositionality, the ability to combine meaningful elements into novel sequences that convey new meanings (de Boer et al. 2012; Hockett 1960). Evidence from several studies suggests that great apes show at least some *precursors* of compositionality (Amici et al. 2022), including the ability to combine multiple gestures into sequences (in captivity: Bard 1992; Genty and Byrne 2010; Liebal et al. 2004; Tanner 2004; Tanner and Perlman 2017; Tempelmann and Liebal 2012; Tomasello et al. 1985, 1994; in the wild: Genty and Byrne 2010; Graham et al. 2018; Hobaiter and Byrne 2011b). In great apes, gestures are usually considered to be part of the same sequence when they are produced by the same individual toward the same partner, within a short timeframe (e.g., up to 1 s: Genty and Byrne 2010; Hobaiter and Byrne 2011b; Tanner 2004; up to 5 s: Liebal et al. 2004; Tempelmann and Liebal 2012). However, the number of gestures within a sequence varies considerably across species and studies (captive chimpanzees, *Pan troglodytes*: 1–3 according to Tomasello et al. 1994; 2–39 according to Liebal et al. 2004; wild chimpanzees: 2–5 according to Roberts et al. 2013; 2–11 according to Hobaiter and Byrne 2011b; captive and wild gorillas, *Gorilla gorilla*: 2–8 according to Genty and Byrne 2010, and Tanner 2004; captive orangutans, *Pongo abelii*: 2–8 according to Tempelmann and Liebal 2012).

Moreover, although sequences including at least two gestures are relatively common in apes, their function remains a topic of debate. Initially, researchers considered sequences as syntactic and semantic combinations of gestures (Tanner 2004; Tomasello et al. 1989, 1994), where the initial gesture could also serve as an attention‐getter (Tomasello and Call 2007). Quantitative investigations have so far failed to support this syntactic and semantic hypothesis, casting doubt about the presence of compositionality in primate gestural sequences (see Amici et al. 2022 for a review). However, signal repetitions remain a hallmark of intentional communication, as signalers may use sequences toward unresponsive recipients as a form of persistence (i.e., repeating the same gesture type) and/or elaboration (i.e., using multiple gesture types) to achieve their communicative goals (e.g., Cartmill and Byrne 2007; Townsend et al. 2017). These goals are typically assessed within the “apparently satisfactory outcome” framework, by examining the recipient's response and the signaler's reaction to it: the cessation of gesturing after the recipient's response is taken as evidence that the intended outcome has been achieved, and the recipient's response is considered a proxy of the gesture meaning (Cartmill and Byrne 2010, and Hobaiter and Byrne 2014). Nonetheless, gestural sequences may not necessarily reflect a signaler's attempt to reiterate and/or elaborate the message. Instead, they may result from high emotional arousal that drives signal production in certain contexts (e.g., when recipients are unresponsive; Graham et al. 2019; Liebal et al. 2013; Townsend et al. 2017; see Tanner and Perlman 2017). Crucially, the fact that sequences may be largely triggered by emotional arousal does not imply that they are not intentional, as in humans, arousal and intentionality likely play complimentary roles in sequence production (Graham et al. 2019).

Most studies indicate that primate gestural sequences often consist of redundant repetitions of the same gesture type (rather than sequences made up of different gestures), which mostly occur in conditions of high arousal, such as during playful interactions or toward unresponsive recipients (in captivity: Aychet et al. 2021; Genty and Byrne 2010; Liebal et al. 2004; Tempelmann and Liebal 2012; in the wild: Genty and Byrne 2010; Roberts et al. 2013). In captive orangutans, for instance, gestural sequences are common but mostly consist of repetitions of the same gesture type, and they are produced regardless of whether recipients respond to the initial gesture (Tempelmann and Liebal 2012), suggesting little similarity to human compositional structures. Overall, these findings imply that gestural sequences are unlikely to function as complex syntactic and semantic combinations that enhance the effectiveness of communication, and might be rather used by primates to emphasize rather than modify the meaning of single gestures, in conditions of high arousal (Amici and Liebal 2022; Genty and Byrne 2010; Graham et al. 2020; Hobaiter and Byrne 2011b; Hobaiter et al. 2022; Rendall 2021; Tanner and Perlman 2017; Tempelmann and Liebal 2012).

In line with this perspective, studies comparing the occurrence of gestural sequences across individuals have shown that sequences are more frequently produced by younger and socially less integrated apes and are less likely to be responded to than single gestures (in captivity: Amici and Liebal 2022; Liebal et al. 2004; in the wild: Hobaiter and Byrne 2011b). These results suggest that, as individuals grow older and become more experienced, they communicate more effectively, reducing the frequency of gestural sequences (Amici and Liebal 2022; Hobaiter and Byrne 2011b; Liebal et al. 2004). However, it is also possible that younger individuals produce sequences more frequently than older ones simply because they engage more often in play sessions where, given the high arousal, sequences might be especially common and response rates lower.

Whether sex differences also influence the probability of producing gestural sequences remains unclear. In captive chimpanzees, Liebal et al. (2004) reported that sequences of ten or more gestures were produced by only two males in the group, whereas Amici and Liebal (2022) found that sequences were more likely within dyads including at least one male, as compared to female‐only dyads, although males were not significantly more likely than females to produce gestural sequences. The exact reasons for these sex differences remain unclear and may reflect variation in specific aspects of their behavior (e.g., personality traits, activity patterns, social interactions they engage in), rather than in their communication systems (Graham et al. 2022). One possibility is that these patterns reflect sex differences in chimpanzee personality. Male chimpanzees are reported to score higher than females on aspects of personality interpreted as Dominance, on which the trait of “persistence” loads highly (Weiss & King 2015), operationalized through ratings of individuals' tendency to persist with tasks and continue goal‐directed behavior despite obstacles. If gestural sequences are used to restate or reinforce a message when initial communicative attempts fail to achieve the desired outcome (Cartmill & Byrne 2007; Townsend et al. 2017), then individuals that are generally more persistent, as suggested for males in one study of captive chimpanzees, might be more likely to produce gestural sequences. Moreover, male chimpanzees typically engage more than females in rough‐and‐tumble play, a high‐arousal context (Marley et al. 2022). If sequences are largely triggered by emotional arousal, and if time spent in different contexts is not controlled for, males may appear to produce more sequences than females, simply because they spend more time in these contexts.

In this study, we investigated gestural sequences in Geoffroy's spider monkeys (*Ateles geoffroyi*), a platyrrhine species for which research on gestural communication remains scarce. To date, research on gestures in species other than great apes has been quite limited, especially in their natural habitat. However, spider monkeys provide a valuable model for the study of gestural communication, as their inclusion complements research on apes and allows broader evolutionary inferences about the emergence of specific communication traits. Gestural sequences, for instance, have been described in great apes and other catarrhines, but it remains unclear whether more distantly related taxa, such as platyrrhines, also produce them and, if so, whether these sequences serve similar functions. Investigating gestural communication in platyrrhines can therefore help determine whether gestural sequences represent a widespread feature of primate communication and whether their patterns of use point to shared underlying competencies, such as their potential use in reinforcement or elaboration of signals, and potentially the capacity to combine gestures in compositionally novel ways.

Here, we made the following hypotheses and predictions. First, we hypothesized evolutionary continuity with other primate taxa and expected that, as in other species (e.g., Hobaiter and Byrne 2011b; Liebal et al. 2004; Tempelmann and Liebal 2012), gestural sequences would occur also in spider monkeys, primarily in contexts of high arousal. In particular, we predicted that the odds of producing gestural sequences, as compared to single gestures, would be higher during playful and aggressive contexts, as compared to other contexts such as resting or feeding (Prediction 1). Moreover, we predicted that, as reported in chimpanzees (e.g., Amici and Liebal 2022; Liebal et al. 2004), the odds of producing gestural sequences, as compared to single gestures, would be higher for males than females (Prediction 2), even when controlling for the context in which gestures occur, as males should be more persistent than females. Finally, we predicted that, as in other species (e.g., Amici and Liebal 2022; Hobaiter and Byrne 2011b; Liebal et al. 2004), the odds of producing gestural sequences would be higher for younger than older individuals (Prediction 3), because gestural sequences might reflect communicative inexperience rather than communicative complexity.

## 2 Methods

### 2.1 Ethical Note

The investigation was entirely observational and non‐invasive. We obtained official permits to conduct our research in Mexico from the CONANP (Comisión Nacional de Áreas Naturales Protegidas) and SEMARNAT (Secretaría de Medio Ambiente y Recursos Naturales) and received the consent of the Mayan community of Punta Laguna residing in the protected area where the study took place. Our methods adhered to the American Society of Primatologists (ASP) Principles for the Ethical Treatment of Nonhuman Primates and the Code of Best Practices for Field Primatology by the American Society of Primatologists and conformed to Directive 2010/63/EU on the protection of animals used for scientific purposes.

### 2.2 Study Site and Subjects

We conducted behavioral observations in the Otoch Ma'ax Yetel Kooh protected area, situated in the Yucatan Peninsula, Mexico (20° 38' N, 87° 38' W). This area is characterized by an undisturbed fragment of semi‐evergreen medium forest with trees reaching heights of up to 25 meters, along with successional forest, regenerating forest and various bodies of water (Ramos‐Fernández and Ayala‐Orozco 2003). The area is inhabited by Geoffroy's spider monkeys (*Ateles geoffroyi*), who have been studied in this natural area for over a quarter of a century. The study group was therefore fully habituated to humans, monkeys could be individually recognized using differences in their facial and body traits, and age was determined from long‐term demographic records maintained at the study site (e.g., Aureli and Schaffner 2007; Schaffner and Aureli 2005; Slater et al. 2009).

The group consisted of 54 individuals, including 13 adult females, 10 adult males, 3 subadult females, 1 subadult male, 9 juvenile females, 6 juvenile males, 3 infant females, 8 infant males and 1 infant of unknown sex (see Shimooka et al. 2008, for a definition of the age categories). Out of these 54 monkeys, 45 (13 adult females, 9 adult males, 2 subadult females, 1 subadult male, 9 juvenile females, 6 juvenile males, 1 infant female and 4 infant males; see Table S1 in Supporting Material) were observed as focal subjects during the study (see below). We excluded the other 9 individuals either because they were born after the study had started (*N* = 3), or because they were still infants that did not detach from their mother's body (*N* = 4), or because they appeared infrequently and sporadically during observations (i.e., they were observed less than 40 min each during focal observations, see below), despite belonging to the main group (*N* = 2). However, all group members could be considered recipients of gestures given by focal individuals.

### 2.3 Data Collection

The first two authors collected data during a 6‐month period (from October 2022 to March 2023), 5 days a week, from 6:30 a.m. to 2:00 p.m, after an initial 2 weeks of training and practice. During data collection, the first two authors conducted 10‐min focal animal samples for a total of 1119 focal samples (mean ± SD: 3.7 ± 1.14 h per focal animal). They selected focal subjects based on a list that we prepared daily, giving priority to focal subjects that had been more rarely observed. One observer used CyberTracker software to enter the data on a tablet, whereas the other observer dictated the data and video‐recorded the focal subject with a camera (Panasonic FULL‐HD HC‐V180), maintaining a wide field of view to also record the behavior of the recipient of a gesture (i.e. the monkey to which a gesture was directed).

### 2.4 Data Coding

At the end of data collection, the first author watched the videos to identify and code all visible gestures, and for each gesture, she further linked information extracted from the video to information recorded with Cybertracker (e.g., to extract group composition, context and other details that might not have been captured in the video: see below for the information extracted). She identified gestures by following the ethogram for spider monkeys (Villa‐Larenas et al. 2024), which lists 45 different gesture types across different modalities (see Table S2 in Supporting Material, for a complete list of gesture types and definitions). We considered a gesture to be any discrete physical movement of the limbs or the head, or any body posture, that was (i) mechanically ineffective and (ii) clearly directed to one (or more) specific recipient(s). We considered movements to be mechanically ineffective when they did not produce the desired outcome by mere physical force (i.e., means‐end dissociation), but which were performed with controlled force to influence the recipient's behavior, rather than directly causing the outcome. Although some gestures such as Grab (see Table S2 in Supporting Material) imply the application of some physical force, we considered them gestures as they were typically produced with controlled force, did not directly produce the outcome (e.g., recruiting partners into joint activities), and have often been included in primate gestural repertoires reported for other species (e.g., Genty et al. 2009; Hobaiter and Byrne 2011a).

In this study, we did not verify goal‐directedness or intentionality for each gesture, but rather relied on prior evidence (Villa‐Larenas et al. 2024) showing that the gesture types included in the ethogram were produced in a goal‐directed, intentional way by members of the study group in the vast majority of cases. This means that, typically, the gestures we included in this study implied the accomplishment of a specific goal (i.e., they usually elicited a particular behavioral response from the recipient), were intentional (as they generally co‐occurred with response‐waiting, i.e., the signaler looked at the recipient and paused after the gesture, awaiting a response), and often involved persistence (i.e., the gesture was repeated if the expected response was not obtained) or elaboration (i.e., the initial gesture was followed by one or more different gesture types to achieve the intended outcome; see Bourjade et al. 2020; Genty et al. 2009; Hobaiter and Byrne 2011a, 2017; Pika 2008; Tomasello and Call 2007; Tomasello et al. 1985, 1994; Villa‐Larenas et al. 2024).

Whenever a gesture occurred, we coded: (i) its type, (ii) the identity of the monkey producing the gesture (i.e., the signaler), (iii) the identity of the monkey to which it was directed (i.e., the recipient), and (iv) the context in which it was produced (i.e., aggression, feeding/foraging, play, resting, sexual behavior, affiliative interaction, traveling; see Villa‐Larenas et al. 2024). We also recorded whether recipients responded to the gesture (i.e., if there was a change in their behavior and/or a shift in gaze toward the signaler within 5 s of the gesture's end). Potential responses included a broad range of behaviors, including gestures, vocalizations and actions such as initiating play, approaching or leaving (see Villa‐Larenas et al. 2024, for a complete list). However, we did not have a sufficiently large independent dataset to quantitatively determine, for each gesture type, which responses signalers might consider satisfactory (i.e., in line with the signaler's goals), as 11 of the 22 gesture types observed as single gestures only occurred once or twice. Therefore, we conducted no analyses on the recipients' response to define gestural meaning (see Hobaiter and Byrne 2014).

We considered gestures to belong to the same sequence if they were produced by the same signaler, toward the same recipient, in the same context and with less than 3 s elapsing between the onset of two consecutive gestures (see Liebal et al. 2004). We selected a 3‐second timeframe based on preliminary observations on the same group of spider monkeys (i.e., most responses to single gestures happened 3–5 s after the end of the gesture, and rapid‐fire strings of gestures were rare). This timeframe is in line with previous studies on other primate species that used a timeframe between 1 and 5 s (e.g., Genty and Byrne 2010; Liebal et al. 2004), although we are aware that setting a priori temporal timeframes, as typically done in studies on primate communication, might have important limitations (see Amici et al. 2022). Gestural sequences were never interrupted (nor followed) by a signal produced by the receiver, so that the sequences we observed were never considered to be part of a “conversation”.

To assess inter‐observer reliability, the second author, who was not blind to the hypotheses, re‐coded 24% (i.e., 75/307) of the events in which either a gesture or a gestural sequence was produced. Inter‐observer reliability was very good for all the variables coded (i.e., Cohen's *k* for gesture type: *k* = 0.91; number of gestures produced during the event: *k* = 0.92; modality of the event: *k* = 0.98; context: *k* = 0.98; whether the recipient was attentive: *k* = 0.87; whether the event was responded: *k* = 0.92; response triggered: *k* = 0.98; all *p* < 0.001).

### 2.5 Statistical Analyses

We conducted statistical analyses using R (R Core Team 2020). First, we used binomial tests to assess whether, in each context, the number of sequences produced, out of all gestural events (i.e., single gestures and sequences), was different from what expected if there were no differences across contexts. Second, we ran a binary logistic mixed model (Baayen et al. 2008) with the glmmTMB package (Brooks et al. 2017). We prepared our dataset entering one line for each gesture or gestural sequence (*N* = 307). We specified the signaler's sex, the signaler's age class (i.e., infant, juvenile, subadult, adult), the signaler's and recipient's identities, observation day, context, and whether it was a single gesture or a sequence (i.e., our response variable). If any of this information was missing (e.g., unclear recipient identification), we excluded those data points (*N* = 49) before running our model (*N* = 258).

We used this dataset to run a single full model with a binomial distribution, testing whether the log odds of producing a sequence (1) or a single gesture (0) varied depending on the following test predictors (which were all included in the same full model): context (Prediction 1), signaler's sex (Prediction 2) and signaler's age class (Prediction 3). Moreover, we included observation day, signaler's and recipient's identities as random factors. To avoid convergence problems, we grouped feeding/foraging and resting, before running the model, because we only had 6 instances of gestures or sequences produced in a feeding/foraging context. In the model, we did not include any interaction between the test predictors, because we did not have any reason to predict that sequences would be more likely for some combinations of the predictors (e.g., we expected sequences to be overall more commonly produced in males than females, and in younger than older individuals, but not only in younger males).

We checked model assumptions with the “performance” (Lüdecke et al. 2021) and “DHARMa” packages (Hartig 2022), and found no evidence of issues with convergence, multicollinearity (max VIF: 2.23; Miles 2005), or dispersion. We ran a likelihood ratio test to compare the full model and the null model, which was identical to the full model but without test predictors. If the full model was significantly better than the null one (i.e., *p* ≤ 0.05, two‐tailed; ΔAIC > 2), we used the drop1 function to test the significance of each test predictor, by dropping a single model term at a time and comparing the reduced and full models using likelihood ratio tests. In case of a significant categorical predictor with more than two categories, like context, we used the emmeans package to assess the estimates of each predictor level and conduct post‐hoc pairwise comparisons using Tukey's adjustment (Lenth 2020).

## 3 Results

### 3.1 Characteristics of Gestures and Gestural Sequences

We recorded a total of 709 gestures during focal observations: 182 were produced as single gestures, and 527 were produced as part of a sequence. We documented a total of 125 sequences, with the longest one consisting of 18 gestures; the majority, however, contained only 2 (*n* = 49), 3 (*n* = 25), or 4 gestures (*n* = 19). The rate of production was therefore 0.98 single gestures and 0.67 sequences per hour. Table S1 in Supporting Material includes the number of single signals and gestural sequences produced by each individual, with the average number of gestures contained in the sequences.

Only eight sequences were observed more than once: grab‐grab pull (*n* = 10), embrace‐pectoral sniff (*n* = 7), push‐present climb (*n* = 3), open mouth‐present genitals (*n* = 2), and slap‐grab‐grab pull (*n* = 2). Grab‐grab pulls were always produced in the context of play, usually by male infants toward other infants (*n* = 7). Embrace‐pectoral sniffs were produced in different contexts, usually by adults (*n* = 4) or subadults (*n* = 1), toward other adults (*n* = 4). Push‐present climbs were always produced by female adults toward infants, while traveling (*n* = 3). The other three sequences that occurred more than once were simple repetitions of the same gesture types (i.e., grab pull, present grooming, open mouth).

**Figure 1:**
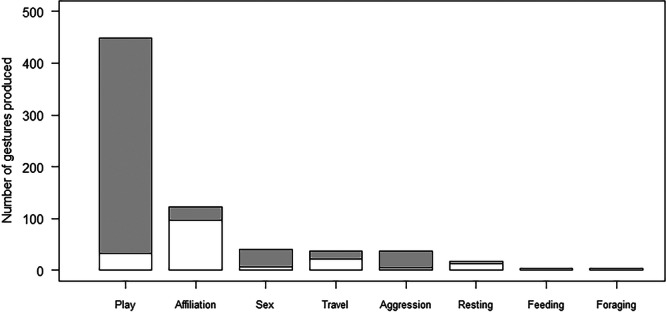
Number of gestures produced as single gestures (in white) or within a sequence (in gray) by spider monkeys, throughout our study, as a function of the context in which they took place.

Gestures were produced in many different contexts, either as singly or in sequences (Table 1; Figure 1). During play, 73% of gestural events (defined as single gestures or sequences) consisted of sequences (*n* = 86), 64% during aggression (*n* = 9), 59% in a sexual context (*n* = 10), 27% when traveling (*n* = 8), 13% when resting (*n* = 2), 9% in affiliative interactions (*n* = 10), and none when feeding and when foraging. Compared to expectations based on the whole dataset (overall odds = 0.70), the odds of producing gestural sequences rather than single gestures were approximately 3.9 times higher during play (odds = 2.70), 2.5 times higher during aggression (odds = 1.78) and 2.0 times higher in a sexual context (odds = 1.44), whereas the odds of producing gestural sequences rather than single gestures were only half as likely during traveling (odds = 0.37), 0.2 times as likely during resting (odds = 0.15) and 0.1 times as likely during affiliative interactions (odds = 0.10). Binomial tests further showed that, as compared to the overall percentage of sequences out of all gestural events (i.e., 41%), sequences were more common than expected during play (*n* = 118, Cohen's *h* = 0.66; *p* < 0.001), and less common than expected during resting (*n* = 15, Cohen's *h* = −0.64; *p* = 0.034) and affiliative interactions (*n* = 107, Cohen's *h* = −0.77; *p* < 0.001), suggesting a more common use of sequences during play.

**Table 1:** Results of the generalized linear mixed model run, with estimates, standard errors (SE), confidence intervals (CIs), likelihood ratio tests (LRT), degrees of freedom (df), and *p* values for each test predictor (marked with an asterisk when significant, i.e. when *p* ≤ 0.05), with the reference category in parentheses.

FULL MODEL: Log odds of producing gestural sequences rather than single gestures ~ context + signaler's sex + signaler's age + (1| signaler) + (1|recipient) + (1|observation day)						
TEST PREDICTORS	Estimate	SE	2.5% to 97.5% CIs	*LRT*	*df*	*p*
Intercept	0.74	0.69	−0.61 to 2.09	—	—	—
Context (feeding/foraging, resting)	−2.98	1.00	−4.93 to ‐1.03	86.04	5	< 0.001*
Context (play)	0.55	0.72	−0.86 to 1.96			
Context (sexual interactions)	−0.38	0.91	−2.15 to 1.40			
Context (other social interactions)	−2.95	0.77	−4.46 to ‐1.43			
Context (traveling)	−1.73	0.79	−3.29 to ‐0.18			
Signaler's sex (male)	0.12	0.38	−0.63 to 0.88	0.10	1	0.748
Signaler's age (infant)	−0.33	0.54	−1.40 to 0.73	0.78	3	0.855
Signaler's age (juvenile)	−0.22	0.48	−1.16 to 0.72			
Signaler's age (subadult)	−0.74	1.20	−3.09 to 1.61			

### 3.2 Odds of Producing Sequences

**Figure 2:**
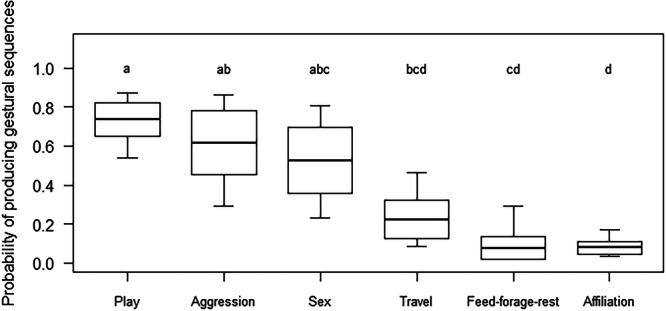
Probability that the gestural event produced was a gestural sequence, rather than a single gesture, as a function of context. The thick lines of the box plots represent the mean probabilities, which were obtained by back‐transforming the odds estimated by the fitted model. The ends of the boxes represent the estimated standard errors, and the ends of the whiskers represent the 95% confidence intervals. Different letters indicate statistically significant differences between contexts based on pairwise comparisons of estimated marginal means: contexts sharing at least one letter do not differ significantly from each other.

The full model significantly differed from the null model (GLMM, *χ* ^2^ = 106.84, *df* = 9, *p* < 0.001; ΔAIC = 88.84), with context having a significant effect on the odds that the focal subject produced a gestural sequence rather than a single gesture (*p* = 0.001; Table 1; Figure 2). In particular, post‐hoc tests showed that sequences were more likely produced during play, as compared to when individuals were traveling (*p* = 0.006), feeding, foraging or resting (*p* < 0.001), or engaging in affiliative interactions other than play (*p* < 0.001). Sequences were also more likely to be produced in aggressive contexts than during feeding, foraging or resting (*p* = 0.033) or during affiliative interactions other than play (*p* = 0.002), and they were also more likely to be produced during sexual contexts as compared to affiliative interactions other than play (*p* = 0.005). Post‐hoc tests revealed no other differences (play‐aggression: *p* = 0.974; play‐sexual behavior: *p* = 0.787; aggression‐traveling: *p* = 0.245; aggression‐sexual behavior: *p* = 0.998; sexual behavior‐traveling: *p* = 0.515; sexual behavior‐feeding, foraging or resting: *p* = 0.081; traveling‐feeding, foraging or resting: *p* = 0.714; traveling‐affiliative interactions: *p* = 0.305; feeding, foraging or resting‐affiliative interactions: *p* = 1.000). Neither of the other test predictors (i.e., signaler's sex and age class) had a significant effect on the odds of producing a gestural sequence rather than a single gesture.

## 4 Discussion

Our study provides an initial description of gestural sequence use in a wild group of platyrrhines, Geoffroy's spider monkeys. As predicted, we found evidence that spider monkeys, like many other primate species, produced both gestural sequences and single gestures toward conspecifics, with production rates of approximately 0.67 sequences per hour and 0.98 single gestures per hour. As in other primate species, the odds of producing gestural sequences rather than single gestures were higher during play and aggression (in line with Prediction 1), and during sexual interactions, compared to other contexts. However, the odds of producing gestural sequences rather than single gestures did not depend on the signaler's sex or age (in contrast to Predictions 2 and 3).

In this study, the majority of gestures produced by spider monkeys were incorporated into sequences, which mostly consisted of between 2 and 4 gestures (and up to 18) individual gestures. In the last decade, few studies have reported the existence of gestural sequences in species other than apes. In catarrhines, gestural sequences have been described in captive olive baboons (*Papio anubis;* Molesti et al. 2019), wild bonnet macaques (*Macaca radiata*; Gupta and Sinha 2019) and captive red‐capped mangabeys (*Cercocebus torquatus*; Aychet et al. 2021). Our study therefore expands these findings by providing evidence that gestural sequences are also used by platyrrhines, suggesting that the ability to combine gestures into sequences might be evolutionary more ancient than previously thought. However, caution is needed when comparing results across studies, as methodologies and operational definitions often differ. For instance, the criterion for considering gestures as part of the same sequence was not specified in studies on olive baboons (Molesti et al. 2019) and bonnet macaques (Gupta & Sinha 2019), whereas in red‐capped mangabeys, gestures were treated as belonging to the same sequence when separated by up to 8 s (Aychet et al. 2021). Such definitional differences limit data comparability and highlight the need for more objective, standardized criteria to define sequences across species. In this regard, future work should standardize procedures, by for instance defining general rules to be applied consistently across taxa, and then, based on these rules, derive *a posteriori* temporary cut‐off points specific to each taxon (similar to Aychet et al. 2021), rather than adopting *a priori* thresholds from the literature (see Amici et al. 2022 for discussion).

Largely in line with Prediction 1 and with literature on other species (Aychet et al. 2021; Bard et al. 2014; Genty and Byrne 2010; Liebal et al. 2004; Tanner and Perlman 2017; Tempelmann and Liebal 2012), we found that the odds of producing gestural sequences rather than single gestures were higher in contexts likely associated with high arousal, and particularly during playful, aggressive and sexual interactions. With regard to play, some authors suggest that play provides a safe context within which primates can flexibly experiment with different forms of communication, including extended gestural sequences (Gibson and Peterson 2002; Kohler 2003; Pellis and Pellis 2007; 2009). In our study, however, the odds of producing sequences rather than single gestures was also higher in aggressive contexts, compared to others, although aggressive interactions do not appear to provide a safe opportunity for primates to unfold their communication skills. These findings suggest that, in spider monkeys, sequences are unlikely to result from the freedom to experiment provided by the safe context of play, but rather may depend on the higher arousal that signalers may experience in specific contexts. To confirm this hypothesis, future research should incorporate physiological measures of emotional arousal during the production of gestures and gestural sequences across contexts. In the wild, thermo‐imaging cameras may represent a promising non‐invasive tool for such investigations (Nieuwburg et al. 2021; Travain et al. 2021).

Out of the 125 sequences observed during this study, only three (i.e., embrace‐pectoral sniff, push‐present climb, grab‐grab pull) occurred more than twice and were not mere repetitions of the same gesture type. In literature, embraces (i.e., monkeys wrap one or two arms around the recipient's back or shoulder, while facing each other) and pectoral sniffs (i.e., monkeys orient their face and nose toward the recipient's chest‐axilla; Schaffner and Aureli 2005; Villa‐Larenas et al. 2024) are indeed described as species‐specific affiliative gestures that often occur concurrently (Rondinelli and Lewis 1976; Schaffner and Aureli 2005). Both embraces and pectoral sniffs are thought to facilitate the regulation of social relationships in spider monkeys, by reducing the risk of aggression in contexts that are likely to be associated with tension (Schaffner and Aureli 2005; Schaffner et al. 2012), like subgroup fusion events and infant handling (Aureli and Schaffner 2007; Rondinelli and Lewis 1976; Schaffner and Aureli 2005; Slater et al. 2009). By implying physical contact and exposing vulnerable body parts to other group members, however, embraces and pectoral sniffs can also entail risk (Schaffner and Aureli 2005). Therefore, their co‐occurrence may be adaptive to increase the likelihood of clearly conveying the sender's affiliative intention and prevent aggressive escalations in potentially dangerous contexts.

With regards to push‐present climb, there is no literature we are aware of on spider monkeys, but it seems plausible that these gestures co‐occur because they may serve a complimentary function, to attract the recipient's attention before facilitating climbing onto the signaler's body. Unsurprisingly, these sequences were always produced by mothers toward infants, although not necessarily theirs. Finally, grab and grab pull are defined in a very similar way in literature, as the signaler holding the hand firmly closed over the recipient's body, but also exerting some force to move the recipient from their position (e.g., in spider monkeys: Villa‐Larenas et al. 2024; in chimpanzees: Hobaiter and Byrne 2011a). There are therefore at least two reasons why grab and grab pull may often co‐occur. First, they might serve a very similar function and thus often be used concurrently in the same situation (e.g., by infants in a play context). Second, top‐down classifications might have wrongly considered grab and grab pull as two different gesture types, whereas they may actually represent simple variations of the same one, such that their co‐occurrence reflects a repetition rather than a sequence of different gesture types. To address this issue, it will be essential in the future to use more objective bottom‐up approaches to identify gesture types (see Grund et al. 2024; Mielke et al. 2023).

Across individuals, the odds of producing gestural sequences rather than single gestures did not depend on the signaler's sex or age, in contrast to our Predictions 2 and 3. These findings are also in contrast to literature on great apes, which has provided some evidence that gestural sequences are produced more often by males (Amici and Liebal 2022; Hobaiter and Byrne 2011b; Liebal et al. 2004) and younger individuals (Amici and Liebal 2022; Hobaiter and Byrne 2011b; Liebal et al. 2004), than by female and older monkeys. One possibility is that these differences reflect a fundamental difference between great apes and other primate species. Notably, male and female spider monkeys also show no differences in the use of different gestural modalities (Villa‐Larenas et al. 2024). Alternatively, inter‐individual variation in apes may simply reflect variation in other aspects of their behavior, such as their activity patterns (Graham et al. 2022), and once variation in individual time budgets is controlled for, as in the present study, patterns across species may prove more similar than currently suggested.

Our study faced several limitations. First, it was based on limited observations (i.e., 709 gestures and 125 sequences), so that additional observational effort might allow detecting other recurrent sequences with different properties. However, some studies in great apes have also relied on a comparable number of sequences (e.g., Liebal et al. 2004). Second, our study relied on a top‐down categorization of gesture types and sequences (i.e., assigning gestures to categories predefined by the authors), including the timeframe we used to define sequences. Although they have been seldom used in literature (Bard et al. 2019; Grund et al. 2024; Mielke et al. 2023), finer‐grained classifications based on bottom‐up approaches (i.e., data‐driven clustering of gestures based on similar observable features, such as hand shape and movement trajectory) might allow a better identification of gesture types and sequences and provide different results (Amici et al. 2022). Third, the study of gestural sequences would surely benefit from a multimodal approach, as it cannot be excluded that monkeys rather rely on multicomponent and multimodal combinations of signals to convey novel meanings (Amici et al. 2022). Future studies should address this gap by evaluating whether and how spider monkeys combine gestures, vocalizations and/or facial expressions, and how recipients respond to these sequences. In arboreal species like spider monkeys, however, the observation of facial expressions will likely posit serious challenges, due to the difficulty of continuously monitoring the face of individual subject in the canopy. Fourth, future studies could explore how the interaction of multiple variables (e.g., signaler's and receiver's characteristics) predicts the use of gestural sequences, as this approach may capture more nuanced dynamics of gestural communication that are undetected with our approach. For example, the goal behind an interaction between infants may be entirely different from that between adults and infants. In the future, it will be important to account for these combinations in the data analysis to ensure more accurate interpretations of gestural communication. Finally, to clarify the communicative function of sequences, future studies should systematically examine (i) recipients' responses to single gestures versus gestural sequences, as this is central to inferring signal meaning and assessing compositionality (see Amici et al. 2022); and (ii) signalers' reaction to recipients' responses, which may reveal whether sequences serve as persistence or elaboration strategies.

Overall, our results provide a novel contribution to the study of gestural sequences in platyrrhines. We showed that sequences were part of the gestural repertoire of wild spider monkeys and that their occurrence is consistent with them driven by emotional arousal, as the odds of producing sequences rather than single gestures were higher in playful, aggressive and sexual contexts. Although we did not assess persistence, elaboration. or compositionality, the fact that sequences were more likely to occur in high‐arousal contexts suggests that they were primarily driven by emotional arousal and less likely to serve elaborative or compositional functions (Cartmill and Byrne 2007; Townsend et al. 2017; Amici et al. 2022), although this possibility cannot be yet completely ruled out. Overall, our findings affirm the value of extending studies of gestural communication to a wider range of primate species (Liebal et al. 2022) and confirm spider monkeys as a further valid model for understanding the evolution of primate gestural communication.

## Author Contributions

**Eva Corral:** investigation, writing – original draft, writing – review and editing, formal analysis, visualization. **Sara Cardoso Rodriguez:** investigation, writing – review and editing. **Katja Liebal:** conceptualization, writing – review and editing, methodology, project administration, resources. **Miquel Llorente:** conceptualization, methodology, writing – review and editing; resources, supervision. **Federica Amici:** conceptualization, writing – original draft, methodology, visualization, writing – review and editing, formal analysis, project administration, supervision, resources.

## Conflicts of Interest

The authors declare no conflicts of interest.

## Supporting information

Supporting File

## Data Availability

The data and script used to analyze the current dataset are available as Supporting Information.
