# Supplementary material for: Gestural Sequences in Wild Spider Monkeys (Ateles geoffroyi)
Source: Am J Primatol. 2026 Apr 27;88(4):e70156. doi: 10.1002/ajp.70156 (PMC13113221; doi:10.1002/ajp.70156)
Supplement: Supplementary file 1 — Supporting File [file AJP-88-e70156-s001.docx]

**TITLE:** Gestural sequences in wild spider monkeys (*Ateles geoffroyi*)

**Table S1.** List of study subjects, including their sex and age class, the number of single gestures produced during the study, the number of gestural sequences, and the number of gestures (average ± standard deviation) included in the sequences (the cell is blank if the subject produced no sequences, and contains no standard deviation if the subject only produced one sequence).

| **Subject** | **Sex** | **Age class** | **Number of single gestures** | **Number of gestural sequences** | **Number of gestures in a sequence** |
| --- | --- | --- | --- | --- | --- |
| Alamo | Male | Infant | 3 | 10 | 3.3 ± 1.8 |
| Antena | Female | Adult | 8 | 3 | 3.0 ± 1.7 |
| Apolo | Male | Adult | 4 | 3 | 3.0 ± 1.0 |
| Aura | Female | Juvenile | 4 | 4 | 8.8 ± 6.6 |
| Bekech | Female | Subadult | 6 | 1 | 2.0 |
| Boxhuevo | Male | Adult | 1 | 2 | 5.5 ± 4.9 |
| Braga | Female | Juvenile | 2 | 3 | 3.7 ± 2.1 |
| Cacao | Male | Infant | 6 | 9 | 6.0 ± 4.3 |
| Canela | Female | Juvenile | 4 | 2 | 5.5 ± 3.5 |
| Chaac | Male | Juvenile | 3 | 0 |  |
| Chikich | Female | Juvenile | 1 | 3 | 3.0 ± 1.7 |
| China | Female | Adult | 3 | 0 |  |
| Covid | Male | Infant | 12 | 10 | 3.4 ± 2.1 |
| Digit | Male | Adult | 4 | 7 | 4.6 ± 2.1 |
| Erbse | Male | Adult | 0 | 0 |  |
| Eulogio | Male | Adult | 1 | 0 |  |
| Fabrizio | Male | Juvenile | 2 | 4 | 8.3 ± 4.2 |
| Ikil | Female | Adult | 0 | 1 | 5.0 |
| Joane | Female | Adult | 7 | 0 |  |
| Juan | Male | Adult | 1 | 1 | 2.0 |
| Lola | Female | Adult | 7 | 0 |  |
| Luna | Female | Juvenile | 6 | 3 | 4.7 ± 3.1 |
| Luz | Female | Juvenile | 4 | 6 | 3.5 ± 0.5 |
| Mandibula | Female | Adult | 7 | 2 | 2.0 ± 0.0 |
| Marcos | Male | Adult | 0 | 0 |  |
| Marylin | Female | Adult | 6 | 3 | 2.0 ± 0.0 |
| Mich | Female | Adult | 7 | 0 |  |
| Nacho | Male | Adult | 3 | 2 | 2.5 ± 0.7 |
| Nit | Female | Subadult | 1 | 0 |  |
| Pancha | Female | Adult | 7 | 5 | 2.4 ± 0.5 |
| Pekin | Male | Juvenile | 8 | 12 | 5.0 ± 4.4 |
| Poncho | Male | Juvenile | 1 | 3 | 5.3 ± 4.0 |
| Puma | Male | Juvenile | 3 | 5 | 3.0 ± 1.0 |
| Rwanda | Female | Adult | 3 | 3 | 2.7 ± 0.6 |
| Sacbe | Female | Juvenile | 5 | 0 |  |
| Sancho | Male | Adult | 2 | 1 | 2.0 |
| Selva | Female | Infant | 2 | 3 | 6.3 ± 6.7 |
| Sol | Male | Infant | 2 | 1 | 2.0 |
| Tanga | Female | Adult | 7 | 0 |  |
| Valentin | Male | Subadult | 2 | 0 |  |
| Veronica | Female | Adult | 7 | 1 | 3.0 |
| Voldemort | Male | Juvenile | 4 | 6 | 3.3 ± 2.3 |
| Xibalba | Female | Adult | 6 | 1 | 2.0 |
| Yalit | Female | Juvenile | 8 | 0 |  |
| Yuli | Female | Juvenile | 2 | 5 | 5.6 ± 3.2 |

**Table S2.** List of gesture types observed, with their definition.

| **Gesture** | **Definition** |
| --- | --- |
| Arm shake | The actor shakes his arm, repeatedly moving it back and forth |
| Arm wrapping | The actor wraps one arm around the recipient’s back, who also wraps the other’s back, while maintaining physical contact and aggressively facing forward toward a third party |
| Beckon | The actor moves his hand in an upward sweep, from the elbow or wrist toward himself |
| Big loud scratch | The actor exaggeratedly scratches his own body with strong scratching movements |
| Bipedal stance | The actor has a bipedal posture, often holding arms out laterally and turning the back to the recipient |
| Bite | The actor gently bites the recipient's body with his lips or teeth |
| Body shake | The actor repeatedly shakes his whole body in the direction of the recipient |
| Bow | The actor bends forward from the waist, while standing upright |
| Dangle | The actor hangs from a branch above another individual, using one or both arms or the tail |
| Dangle shake | The actor hangs from a branch above another individual, using one or both arms or the tail, while (repeatedly) shaking his body |
| Embrace | The actor wraps one or both arms around the recipient’s back or neck, while maintaining physical contact and facing each other |
| Embrace tail | The actor wraps his tail around the recipient's tail while facing each other, so that the two tails are intertwined with each other |
| Frontal threat | The actor leans in the direction of the recipient, extending his back while being supported by one lower limb and one upper limb (sometimes also the tail), whereas the other limbs are free |
| Gallop | The actor makes exaggerated running movements, so that the contact of his hands and feet on the branches is clearly audible |
| Grab/ Grab hold | The actor holds his hand firmly closed over the recipient's body (if longer than 2 sec, it is considered a Grab hold) |
| Grab pull | The actor holds his hand firmly closed over the recipient's body, but force is exerted to move the recipient from his position |
| Hand on | The actor places his hand(s) on the recipient (typically the palm), and maintains contact for at least 2 sec |
| Hand shake | The actor repeatedly moves the hand back and forth, from the wrist |
| Head butt | The actor briefly and firmly pushes his head towards the recipient's body |
| Head shake | The actor shakes his head, repeatedly moving it back and forth |
| Hit other | The actor moves his whole arm or tail, leading to a brief but hard contact of the closed fist or tail with the recipient's body |
| Leaf clipping | The actor tears strips of a leaf/leaves off with the teeth or mouth, which he holds in his hand, thereby producing a flashy sound |
| Object shake | The actor shakes an object, repeatedly moving it back and forth, while looking at the recipient |
| Open mouth | The actor opens his mouth, by lowering the lower lip and raising the upper lip with short repetitive movements, and teeth may become visible |
| Pectoral sniff | The actor places his head in the area of the recipient’s chest-axilla |
| Pirouette | The actor turns around the vertical axis of his body |
| Poke | The actor firmly and briefly pushes one or more fingers on the recipient's body |
| Pounce | The actor moves through the air to land quadrupedally on the recipient's body |
| Present climb | The actor (usually a mother) extends his arm or leg to an immature to facilitate climbing onto his body |
| Present genitals | The actor (usually a female) approaches the recipient (usually a male) from behind, exposing the swelling or anus to the recipient's attention |
| Present grooming | The actor exposes an area of his body to the recipient's attention, as if soliciting grooming |
| Pull tongue | The actor sticks out his tongue in the direction of the recipient |
| Push | The actor puts the palm of his hand in contact with the recipient's body, exerting force in an attempt to displace the recipient, sometimes toward a specific point or direction |
| Roll over | The actor rolls onto his back exposing his stomach, usually while repeatedly making arm and/or leg movements |
| Shake hands | The actor grasps the recipient's hand with his own hand, and then makes small repeated movements back and forth from the wrist |
| Slap | The actor moves his arm from the shoulder, so that the hand or fingers come in short but hard contact with the recipient |
| Somersault | The actor curls his body into a compact position on the floor and rolls forward, so that his feet go over his head before he returns to a seated position |
| Stiff walk | The actor walks quadrupedally with slow exaggerated movements |
| Stomp | The actor vertically lifts one foot/both feet and brings the sole into brief audible contact with the surface on which it rests (i.e. ground or branch) |
| Tandem walk | The actor puts his arm over the recipient's body and both walk forward while maintaining this position, often in a play context to recruit other players |
| Tap other | The actor moves his arm from the wrist or elbow, leading to a repetitive firm short contact between his fingers and the recipient's body |
| Touch | The actor places his hand or fingers or tail on the recipient, maintaining contact for less than 2 sec |

**SCRIPT USED FOR THE DATA ANALYSES**

# LOAD PACKAGES

library(glmmTMB)

library(performance)

library(emmeans)

library(DHARMa)

# LOAD AND CHECK DATA

setwd("C:/....")

xdata=read.table(file="m1.txt", header=T, sep="\t")

str(xdata)

hist(xdata$seq1.noseq0)

# RUN FULL MODEL

res1=glmmTMB(seq1.noseq0 ~ context + sub.sex + sub.age +

(1|sub) + (1|rec) + (1|obs.day), family="binomial", data=xdata)

# CHECK FULL MODEL

check_collinearity(res1)

testDispersion(res1)

# COMPARE FULL AND NULL MODELS

null1=glmmTMB(seq1.noseq0 ~

(1|sub) + (1|rec) + (1|obs.day), family="binomial", data=xdata)

anova(null1, res1, test="Chisq")

# TEST PREDICTOR SIGNIFICANCE

drop1(res1, test="Chisq")

summary(res1)

round(confint(res1),digits=2)

# RUN POST HOC TESTS

emmeans(res1, pairwise ~ context, type='response')

# RUN BINOMIAL TESTS (examples for aggression and sexual context)

p=125/(125+182)=0.41

# aggression

binom.test(9, 14, 0.41)

2*asin(sqrt(9/14))-2*asin(sqrt(0.41))

# sexual

binom.test(10, 17, 0.41)

2*asin(sqrt(10/17))-2*asin(sqrt(0.41))
